# MAGeCK enables robust identification of essential genes from genome-scale CRISPR/Cas9 knockout screens

**DOI:** 10.1186/s13059-014-0554-4

**Published:** 2014-12-05

**Authors:** Wei Li, Han Xu, Tengfei Xiao, Le Cong, Michael I Love, Feng Zhang, Rafael A Irizarry, Jun S Liu, Myles Brown, X Shirley Liu

**Affiliations:** Department of Biostatistics and Computational Biology, Dana-Farber Cancer Institute, Harvard School of Public Health, Boston, MA 02215 USA; Center for Functional Cancer Epigenetics, Dana-Farber Cancer Institute, Boston, MA 02215 USA; Division of Molecular and Cellular Oncology, Department of Medical Oncology, Dana-Farber Cancer Institute, Boston, MA 02215 USA; Broad Institute of MIT and Harvard, 75 Ames Street, Cambridge, MA 02142 USA; Broad Institute of MIT and Harvard, 7 Cambridge Center, Cambridge, MA 02142 USA; McGovern Institute for Brain Research, Department of Brain and Cognitive Sciences, Department of Biological Engineering, Massachusetts Institute of Technology, Cambridge, MA 02139 USA; Department of Statistics, Harvard University, Science Center 715, 1 Oxford Street, Cambridge, MA 02138 USA; Department of Medicine, Brigham and Women’s Hospital and Harvard Medical School, Boston, MA 02215 USA

## Abstract

We propose the Model-based Analysis of Genome-wide CRISPR/Cas9 Knockout (MAGeCK) method for prioritizing single-guide RNAs, genes and pathways in genome-scale CRISPR/Cas9 knockout screens. MAGeCK demonstrates better performance compared with existing methods, identifies both positively and negatively selected genes simultaneously, and reports robust results across different experimental conditions. Using public datasets, MAGeCK identified novel essential genes and pathways, including *EGFR* in vemurafenib-treated A375 cells harboring a *BRAF* mutation. MAGeCK also detected cell type-specific essential genes, including *BCR* and *ABL1*, in KBM7 cells bearing a *BCR-ABL* fusion, and *IGF1R* in HL-60 cells, which depends on the insulin signaling pathway for proliferation.

**Electronic supplementary material:**

The online version of this article (doi:10.1186/s13059-014-0554-4) contains supplementary material, which is available to authorized users.

## Background

The clustered regularly interspaced short palindromic repeats (CRISPR)/Cas9 system is a revolutionary approach for genome editing of mammalian cells [1,2]. In this system, single-guide RNAs (sgRNAs) direct Cas9 nucleases to induce double-strand breaks at targeted genomic regions. The 5′ end of sgRNAs includes a nucleotide sequence of around 20 nucleotides that is complementary to the targeted region. When the double-strand breaks are repaired by non-homologous end-joining (NHEJ), insertions and deletions occur with high frequency, thus efficiently knocking out the targeted genomic loci. The recent development of a lentiviral delivery method has enabled the creation of genome-scale CRISPR/Cas9 knockout (or 'GeCKO') libraries targeting 10^2^ to 10^4^ genes. These libraries allow both negative and positive selection screening to be conducted on mammalian cell lines in a cost-effective manner [3-6]. In CRISPR/Cas9 knockout screens, each gene is targeted by several sgRNAs, and the mutant pool carrying different gene knockouts could be resolved by high-throughput sequencing.

The genome-wide CRISPR/Cas9 knockout technology shows greater promise compared with other loss-of-function screen techniques such as RNA interference (RNAi), because it is able to knockout genes at the DNA level. However, the data generated by these screens pose several challenges to computational analysis. First, studies are often carried out with no or few replicates, which necessitates a proper statistical model to estimate the variance of the read counts and to evaluate the statistical significance of comparisons between treatment and control samples. The observed sgRNA abundance is highly variable in both positive and negative selection experiments (Figure S1 in Additional file 1), and is over-dispersed compared with a Poisson sampling model. (This over-dispersion is similar to the observations from other high-throughput sequencing experiments such as RNA-Seq [7,8]). Second, as different sgRNAs targeting the same gene might have different specificities [9-11] and knockout efficiencies, a robust method is needed to take these factors into account in the aggregation of information from multiple sgRNAs (see Figure S2 in Additional file 1 for an example). Third, depending on different screen libraries and study designs, the read count distributions of the CRISPR/Cas9 knockout screening experiments are different, as positive selection often results in a few sgRNAs dominating the total sequenced reads (Figure S3 in Additional file 1). This calls for a robust normalization of the sequenced reads.

Several existing algorithms, although not specifically designed for CRISPR/Cas9 knockout screens, can be used to identify significantly selected sgRNAs or genes. For example, edgeR [7], DESeq [8], baySeq [12] and NBPSeq [13] are commonly used algorithms for differential RNA-Seq expression analysis. These algorithms are able to evaluate the statistical significance of hits in CRISPR/Cas9 knockout screens, although only at the sgRNA level. Algorithms designed to rank genes in genome-scale short interfering RNA (siRNA) or short hairpin RNA (shRNA) screens can also be used for CRISPR/Cas9 knockout screening data, including RNAi Gene Enrichment Ranking (RIGER) [14] and Redundant siRNA Activity (RSA) [15]. However, these methods are designed to identify essential genes mostly from oligonucleotide barcode microarray data, and a new algorithm is needed to prioritize sgRNAs, as well as gene and pathway hits from high-throughput sequencing data.

We developed a statistical approach called Model-based Analysis of Genome-wide CRISPR/Cas9 Knockout (MAGeCK) to identify essential sgRNAs, genes and pathways from CRISPR/Cas9 knockout screens. We use the term 'essential' to refer to positively or negatively selected sgRNAs, genes or pathways. MAGeCK outperforms existing computational methods in its control of the false discovery rate (FDR) and its high sensitivity. MAGeCK’s results are also robust across different sequencing depths and numbers of sgRNAs per gene. Furthermore, using public CRISPR/Cas9 knockout screening datasets, we demonstrate that MAGeCK is able to perform both positive and negative selection screens simultaneously, and identify biologically meaningful and cell type-specific essential genes and pathways.

## Results and discussion

### Overview of the MAGeCK algorithm

**Figure 1:**
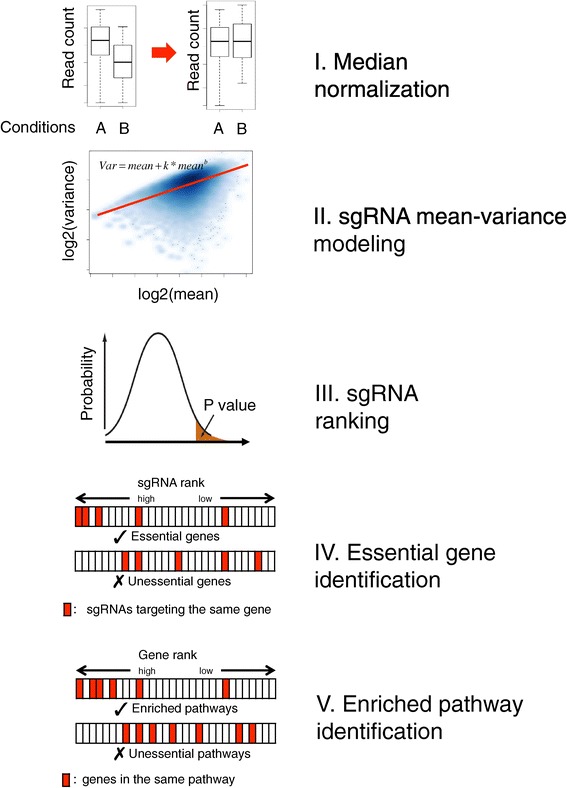
**Overview of the MAGeCK algorithm.** Raw read counts corresponding to single-guided RNAs (sgRNAs) from different experiments are first normalized using median normalization and mean-variance modeling is used to capture the relationship of mean and variance in replicates. The statistical significance of each sgRNA is calculated using the learned mean-variance model. Essential genes (both positively and negatively selected) are then identified by looking for genes whose sgRNAs are ranked consistently higher (by significance) using robust rank aggregation (RRA). Finally, enriched pathways are identified by applying the RRA algorithm to the ranked list of genes.

A schematic of the MAGeCK algorithm is presented in Figure 1. Briefly, read counts from different samples are first median-normalized to adjust for the effect of library sizes and read count distributions. Then the variance of read counts is estimated by sharing information across features, and a negative binomial (NB) model is used to test whether sgRNA abundance differs significantly between treatments and controls. This approach is similar to those used for differential RNA-Seq analysis [7,8,13]. We rank sgRNAs based on *P*-values calculated from the NB model, and use a modified robust ranking aggregation (RRA) algorithm [16] named α-RRA to identify positively or negatively selected genes. More specifically, α-RRA assumes that if a gene has no effect on selection, then sgRNAs targeting this gene should be uniformly distributed across the ranked list of all the sgRNAs. α-RRA ranks genes by comparing the skew in rankings to the uniform null model, and prioritizes genes whose sgRNA rankings are consistently higher than expected. α-RRA calculates the statistical significance of the skew by permutation, and a detailed description of the algorithm is presented in the Materials and methods section. Finally, MAGeCK reports positively and negatively selected pathways by applying α-RRA to the rankings of genes in a pathway.

### CRISPR/Cas9 knockout screen datasets

We examined three recently published CRISPR/Cas9 knockout screen experiments [3,4,6]. The first experiment (or 'ESC dataset') performed negative selection on mouse embryonic stem cells (ESCs) to screen for essential genes. The second experiment (or 'leukemia dataset') [3] performed similar negative selection experiments on the chronic myeloid leukemia cell line KBM7 and the acute promyelocytic leukemia cell line HL-60. The controls for these studies were cells before Cas9 activation. The third experiment (or 'melanoma dataset') [4] was based on one human melanoma cell line (A375), which harbors a V600E mutation in the *BRAF* protein kinase gene. In this study, positive selection was performed to identify genes whose knockouts resulted in resistance to 7-day and 14-day treatment with the BRAF inhibitor vemurafenib (PLX), and the controls were cells treated with dimethyl sulfoxide (DMSO).

### MAGeCK outperforms other methods in detecting significantly selected sgRNAs and genes

We compared MAGeCK with two different categories of methods, including methods for statistical evaluation of high-throughput sequencing read counts using NB models (edgeR and DESeq), and methods originally designed for ranking genes in genome-scale RNAi screens (RIGER and RSA). A summary of the comparisons between MAGeCK and these algorithms is presented in Table 1.

**Table 1:** **A comparison of MAGeCK with existing shRNA/siRNA screening methods: RIGER, RSA, edgeR and DESeq**

	**Methods**	**MAGeCK**	**RIGER [** 14 **]**	**RSA [** 15 **]**	**edgeR [** 7 **] /DESeq [** 8 **]**
**sgRNA ranking**	Ranking method	Negative binomial *P*-value	Signal-to-noise ratio	Fold change	Negative binomial *P*-value
	Statistical evaluation	Yes	No	No	Yes
	Number of samples required in each category	1, prefer more	At least 2	1	1
	Bias towards sgRNAs with smaller read counts^a^	No	No	Yes	No
**Gene ranking**	Ranking method	Robust rank aggregation *P*-value	Kolmogorov-Smirnov *P*-value	Iterative hyper-geometric *P*-value	Not applied to gene ranking
	Permutation	Yes	Yes	No	
	FDR^b^	Low	Low	High	
	Sensitivity in detecting negatively selected genes^c^	High	Low	High	
	Robust against the number of sgRNAs/gene^d^	Yes	No	Yes	

^a^Evaluated in Figure S5 in Additional file 1.

^b^Evaluated in Figure 2a and in Table S1 in Additional file 2.

^c^Evaluated in Figure 2a and in Table S1 in Additional file 2.

^d^Evaluated in Figure 5.

We first compared MAGeCK with edgeR and DESeq. All three algorithms model the high variance of sgRNAs with higher mean read counts (Figure S1 in Additional file 1). The variance models of MAGeCK and DESeq are similar, while edgeR has a lower variance estimation when read counts are low. We also evaluated the FDR of different algorithms by making comparisons between control samples and between replicates of the treatment samples in the ESC and melanoma datasets (there were no replicated treatment samples in the leukemia dataset). Since the CRISPR/Cas9 knockout system should show no difference in selection preference between control samples or between replicated treatment samples, a good method should not detect many significantly selected sgRNAs and genes between these samples. MAGeCK identified fewer significantly selected sgRNAs using the NB model than edgeR and DESeq (see Section A of Supplementary materials in Additional file 1 for more details). The distribution of the calculated *P*-values for all the sgRNAs approximates a uniform distribution (Figure S4 in Additional file 1), which indicates that our model controls the specificity for comparisons where we expect no true positives.

**Figure 2:**
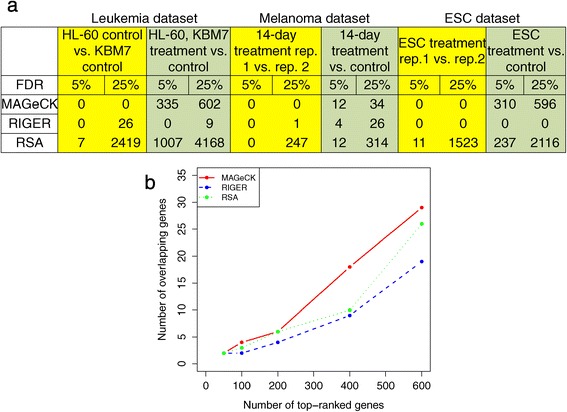
**A comparison of MAGeCK with two other RNA interference screen algorithms, RIGER and RSA. (a)** The numbers of significantly selected genes identified by MAGeCK, RIGER and RSA in different comparisons. For comparisons between control samples or between replicates of the same condition (highlighted in yellow), ideally no significantly selected genes should be detected. Comparisons between treatments and controls are highlighted in green. See Table S1 in Additional file 2 for a complete comparison. **(b)** The overlap of top-ranked genes between CRISPR/Cas9 knockout screening and RNAi screening on the melanoma dataset. The positive screening experiment was performed in the same way as for the melanoma dataset [17], except that pooled shRNA screening was used instead of CRISPR/Cas9 knockout screening.

Next we compared the performance of MAGeCK with two RNAi screening algorithms, RIGER and RSA, at both the sgRNA and gene level. MAGeCK ranks sgRNAs based on the NB *P*-values, while the ranking of RIGER is based on the signal-to-noise ratio. RSA ranks sgRNAs based on their fold change between treatment and controls, but this approach introduces bias towards sgRNAs with fewer read counts (Figure S5 in Additional file 1). At the gene level, RIGER’s sensitivity was lower, and it identified less than 30 significantly selected genes in all datasets, and missed many of the essential genes (for example, ribosomal genes) in two negative screening studies [3,6] (Figure 2a). RSA had low specificity and reported high numbers of genes, even when comparing controls or replicates (Figure S6 in Additional file 1, Table S1 in Additional file 2). In contrast, MAGeCK was able to detect significant genes when comparing treatments with controls, while giving very few false positives when comparing controls or replicates (Figure 2a; Table S1 in Additional file 2).

Finally, we compared the screening results from the melanoma dataset with those from an independent study which used pooled shRNAs to screen PLX-treated A375 cells [17]. We applied MAGeCK, RIGER and RSA to both the CRISPR/Cas9 knockout screens and shRNA screens and checked the consistency of the top-ranked genes (Figure 2b). Although the overall consistency of genes called from the different screens was low (fewer than 5% overlap), MAGeCK always identified more consistent genes than RIGER and RSA at different cutoffs. This shows that MAGeCK can be used for both RNAi screens and CRISPR/Cas9 knockout screens, and that MAGeCK identifies more consensus hits between different screening technologies than other methods (Table S2 in Additional file 2).

### MAGeCK reports robust results with different sequencing depths and different numbers of sgRNAs per gene

**Figure 3:**
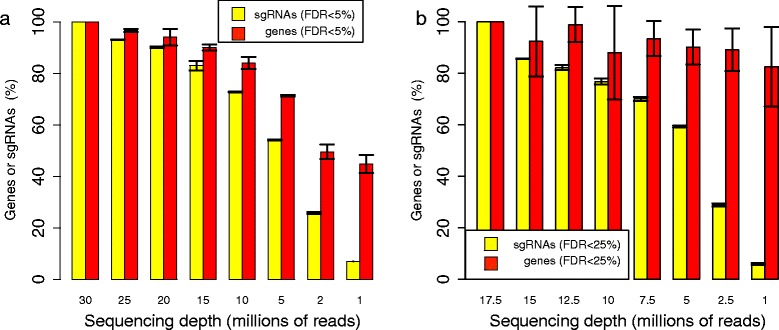
**MAGeCK is robust against sequencing depth and the number of targeting sgRNAs per gene. (a)** The number of significantly selected sgRNAs and genes in the leukemia dataset using various sequencing depths. The maximum sequencing depth for all samples is 30 million. See Materials and methods for sampling details. **(b)** The number of significantly selected sgRNAs and genes in the melanoma dataset using various sequencing depths. The maximum sequencing depth for all samples is 17.5 million. See Materials and methods for sampling details. Error bars represent the standard deviation from three independent sampling experiments.

**Figure 4:**
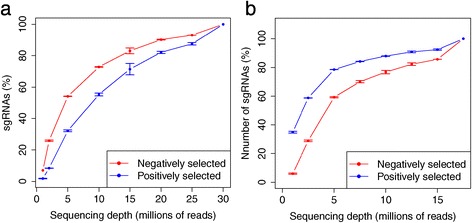
**The number of identified positively and negatively selected sgRNAs at different sequencing depths. (a,b).** The numbers of positively and negatively selected sgRNAs in the leukemia dataset (a) and melanoma dataset (b) under different sequencing depths are shown. The numbers are normalized by the number of identified sgRNAs at the maximum sequencing depths (30 million for the leukemia dataset, 17.5 million for the melanoma dataset).

Both sequencing depth and the number of targeting sgRNAs per gene affect the CRISPR/Cas9 knockout screening experiment outcomes substantially. To study the effect of sequencing depth on performance, we randomly sampled sequencing reads in one negative screening dataset (the leukemia dataset) and one positive screening dataset (the melanoma dataset), and used MAGeCK to identify significantly selected sgRNAs and genes. We compared the numbers of significantly selected sgRNAs and genes that are identified for different numbers of down-sampled reads (Figures 3 and 4; see Materials and methods for more details). At the sgRNA level, less than 10% of the sgRNAs could be detected in the datasets with one million reads (or 3.3% and 5.7% of the reads in the leukemia and melanoma datasets, respectively) compared with the full datasets. At the gene-level, however, MAGeCK could still detect, on average, over 40% and 80% of the genes in the full leukemia and melanoma datasets, respectively. This suggests that the robust rank aggregation approach makes MAGeCK robust to sequencing depth. Interestingly, MAGeCK could detect over 30% of the significantly positively selected sgRNAs in the melanoma dataset using only 1 million reads (Figure 4), a much larger fraction compared with the negatively selected genes in both datasets. This is because the reads corresponding to these sgRNAs dominate the library population (Figure S7 in Additional file 1), and the sequencing depth required to detect positively selected sgRNAs is much less in the positive selection screens.

**Figure 5:**
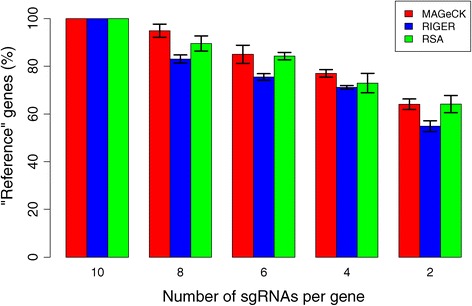
**MAGeCK is robust to the number of targeting sgRNAs per gene.** This figure shows the effect of different numbers of targeting sgRNAs per gene. Each bar indicates the percentage of top-ranked, 'reference' genes that are identified by MAGeCK, RIGER and RSA using different numbers of sgRNAs per gene. 'Reference' genes are those that are in the top 5% of ranked genes in all three methods when using 10 sgRNAs per gene. See Materials and methods for sampling details. Error bars represent the standard deviation from three independent sampling experiments.

We next evaluated the performance of the different algorithms after reducing the number of sgRNAs in a CRISPR/Cas9 knockout screen. The leukemia dataset was used since, on average, >10 sgRNAs were designed to target each gene. As the true essential genes are unknown, we selected 168 'reference' genes that are consistently ranked among the top 5% by all three methods using 10 sgRNAs/gene. We then tested whether the algorithms can detect these 'reference' genes using fewer sgRNAs (Figure 5; see Materials and methods for more details). Both MAGeCK and RSA detected more reference genes than RIGER, and could still identify over 80% of these 'reference' genes with four to six sgRNAs per gene (Figure 5). This suggests that when there are fewer sgRNAs available for some genes, MAGeCK and RSA can still make robust calls.

### MAGeCK identifies known and novel biologically interesting genes and pathways

We applied MAGeCK to the original CRISPR/Cas9 knockout screen studies to identify positively and negatively selected genes and pathways. Genes in pathways from the KEGG (Kyoto Encyclopedia of Genes and Genomes) and REACTOME databases were evaluated for pathway enrichment (Tables S3 to S10 in Additional file 2; Tables S11 to S18 in Additional file 3). In the leukemia and ESC CRISPR/Cas9 knockout screen studies, negatively selected genes were enriched in many fundamental pathways (Tables S9 and S10 in Additional file 2; Tables S10 to S14 in Additional file 3) [3,6]. Pluripotency genes and genes that are well known to be essential for ESC proliferation were also negatively selected, consistent with the observations reported in the original study (Table 2). In the melanoma dataset, the oxidative phosphorylation pathway was negatively selected in the normal condition (treated with 14-day DMSO versus 7-day DMSO), supporting the hypothesis that melanoma cells are addicted to oxidative phosphorylation [18]. Under the PLX treated condition, in addition to the genes that were reported before [4] (Table S7 in Additional file 2), MAGeCK also identified several new positively selected genes (Table 2), such as *CDH13* (FDR = 1.7e-2, ranked 9th out of 17,419) and *PPT1* (FDR = 8.5e-2, ranked 14th out of 17,419). Loss-of-function mutations of *PPT1* cause neuronal ceroid lipofuscinosis and are resistant to apoptosis induction [3-6,19]. *CDH13*, a tumor suppressor that negatively regulates cell growth, is frequently hyper-methylated and contributes to tumorigenesis in melanoma, lung and colorectal cancers [7,8,20,21]. Interestingly, these cancers often harbor a BRAF V600E mutation that can be treated with the BRAF inhibitor PLX, and this mutation is also present in the melanoma cell line used in this CRISPR/Cas9 knockout screen. Our results imply that tumor patients harboring BRAF V600E mutations might have suboptimal response to PLX treatment if their tumors have *CDH13* hypermethylation.

**Table 2:** **Significant positively (and negatively) selected genes and pathways that have experimental support in different comparisons**

**Dataset**	**Comparisons**	**Direction**	**Genes or pathways**	**FDR**	**Rank**	**Experimental support**
Leukemia	HL-60, KBM7, treatment versus control	Positive	*MAP4K3*	0.14	9	[22]
			*EPM2A*	0.14	10	[23]
		Negative	KEGG: ribosome	4.71E-4	1/181	[3]
	HL-60 versus KBM7	Negative	*IGF1R*	1.98E-3	1	[30]
	KBM7 versus HL-60	Negative	*BCR*	1.60E-3	7	[31]
			*ABL1*	1.98E-3	18	
			KEGG: chronic myeloid leukemia	9.00E-4	6/181	
Melanoma	PLX treatment versus control (14 days)	Positive	*CDH13*	0.017	9	[20,21]
			*PPT1*	0.085	14	[19]
			*NF1*, *NF2*, *MED12*, *CUL3*, *TADA1*, *TADA2B*	<0.031	11 (max)	[4]
		Negative	*RREB1*	0.050	1	[25,26]
	PLX treatment versus control (7 days)	Positive	*NF1*, *NF2*, *MED12*, *CUL3*, *TADA1*, *TADA2B*	<0.030	26 (max)	[4]
		Negative	*EGFR*	0.025	6	[28,29]
			REACTOME: *SHC1* events in *EGFR* signaling	0.069	1/676	
			REACTOME: signaling by constitutive active *EGFR*	0.069	2/676	
	DMSO treatment 14 days versus 7 days	Negative	KEGG: oxidative phosphorylation	3.30E-3	2/181	[18]
ESC	ESC versus plasmid	Positive	*TRP53*	0.010	1	[24]
		Negative	KEGG: ribosome	2.83E-4	1/181	[6]
			*NANOG*, *POU5F1*, *RAD51*, *BRCA1*	<0.016	217 (max)	

For other top ranked genes, see Tables S3 to S10 in Additional file 2 and Tables S10 to S18 in Additional file 3.

### MAGeCK allows bi-directional screening and cell-type-specific screening

Although the original leukemia and ESC studies are negative screens and the melanoma study is a positive screen, MAGeCK is also able to perform bi-directional analysis to search for both positively and negatively selected genes simultaneously. This functionality allows MAGeCK to gain biological insights beyond the original screen design. For example, MAGeCK identified several positively selected genes from both negative-selection screens (the leukemia and ESC datasets), and negatively selected genes in the positive-selection screen (the melanoma dataset) (Table 2; Tables S4 and S8 in Additional file 2; Table S12 in Additional file 3). In the leukemia dataset, MAGeCK identified 23 positively selected genes, whose knockout induces cell proliferation. They include *MAP4K3* (FDR = 0.14, ranked 9th out of 7,115), a tumor suppressor kinase in the mitogen-activated protein kinase (MAPK) pathway which induces apoptosis [9-11,22], and *EPM2A* (FDR = 0.14, ranked 10th out of 7,115), another protein phosphatase that negatively regulates cell cycle progression [7,23]. From the ESC dataset, *TRP53*, a mouse ortholog of the human *TP53* tumor suppressor gene [8,24], was ranked first out of the three positively selected genes identified. The negative regulator functions of these genes are consistent with our results that knocking them out confers a selective advantage for cell growth. From the melanoma dataset, MAGeCK only identified one negatively selected gene, *RREB1*, in the 14-day PLX treatment. *RREB1* (FDR = 0.05, ranked 1st out of 17,419) is a transcription factor and a downstream activator in the RAS-RAF signaling pathway [12,25,26], which is closely related to the *BRAF* mutation found in A375 cells [13,27]. Interestingly, MAGeCK also found *EGFR* (FDR = 0.025, ranked 6th out of 17,419) and its associated pathways to be negatively selected in the 7-day PLX-treated samples, implying that PLX-treated cells are more dependent on *EGFR*. Our finding is consistent with recent studies linking ectopic *EGFR* expression in melanoma cells to PLX resistance [14,28] and with the improved efficacy of *BRAF* and *EGFR* combination inhibition in colorectal cancer cells with the BRAF V600 mutation [15,29].

Finally, we applied MAGeCK to identify cell type-specific essential genes and pathways that differ between the chronic myeloid leukemia cell line KBM7 and the acute promyelocytic leukemia cell line HL-60, which are part of the leukemia dataset [3,7,8,13] (Tables S15 to S18 in Additional file 3). MAGeCK identified the KEGG 'chronic myeloid leukemia' pathway as essential in KBM7 (FDR = 9.00e-4, ranked 6th out of 181), correctly distinguishing the cell type differences between KBM7 and HL-60. At the gene level, *IGF1R* (FDR = 1.98e-3, ranked 1st out of 7,115) was found to be specifically essential in HL-60, which is consistent with the observation that an *IGF1R* inhibitor reduces proliferation and induces apoptosis in HL-60 cells [16,30]. In addition, MAGeCK identified *BCR* (FDR = 1.60e-3, ranked 7th out of 7,115) and *ABL1* (FDR = 1.98e-3, ranked 18th out of 7,115) as specifically essential in KBM7, which is consistent with the presence of the *BCR-ABL* fusion in this cell line [3,4,6,31]. The ability of MAGeCK to identify cell type-specific essential genes will be very useful as more CRISPR/Cas9 knockout screening data become publicly available.

## Conclusions

The recently developed genome-scale CRISPR/Cas9 knockout screening technology is a promising tool to select essential genes in mammalian cells. We developed a computational algorithm MAGeCK to reliably identify essential sgRNAs, genes and pathways from CRISPR/Cas9 knockout screens. Compared with existing algorithms that use high-throughput sequencing counts (for example, edgeR, DESeq and baySeq) or RNAi screens (for example, RIGER and RSA) to detect significantly selected sgRNAs and genes, MAGeCK has high sensitivity and a low FDR. It is also robust to different sequencing depths and different numbers of sgRNAs targeting each gene, which will allow more cost-effective CRISPR/Cas9 knockout screening experiments to be performed.

MAGeCK yielded novel biological insights from the re-analysis of three public CRISPR/Cas9 knockout screening datasets. It identified biologically meaningful essential genes and pathways that were missed in the original studies, and found cell type-specific essential genes by comparing CRISPR/Cas9 knockout screens from different cell types. We also demonstrated MAGeCK’s ability to simultaneously identify genes under both positive and negative selection in one dataset. This allowed us to explore new features beyond the original CRISPR/Cas9 knockout screen design, for example, to identify new drug response genes and potential combination therapies (for example, *EGFR* in *BRAF* mutated cancer cells).

Taken together, our results demonstrate that MAGeCK is a useful tool for the computational analysis of CRISPR/Cas9 knockout screens, although our evaluation is based on the limited number of public datasets (and replicates) that are currently available. The mean-variance model of MAGeCK fits the data slightly better than DESeq and edgeR in these datasets, and the MAGeCK algorithm may be further improved as more public CRISPR/Cas9 knockout screening datasets accumulate in the public domain. CRISPR/Cas9 knockout screens that target non-coding regions (for example, long non-coding RNAs, enhancers, microRNAs) will be more challenging, as the number of targeting sgRNAs that can be designed is limited. It is likely that it will be possible to further improve MAGeCK’s algorithm by considering additional factors that may affect the experimental outcome, such as the sequence context and the knockout efficiency of each sgRNA.

## Materials and methods

### The MAGeCK algorithm

MAGeCK is designed to identify positively and negatively selected sgRNAs and genes in genome-scale CRISPR/Cas9 knockout experiments. It consists of four steps: read count normalization, mean-variance modeling, sgRNA ranking and gene ranking.

#### Read count normalization

Suppose there are *N* CRISPR/Cas9 knockout screening experiments performed on a set of *M* sgRNAs, and the read count of sgRNA *i* in experiment *j* is *x*_*ij*_, 1 ≤ *i* ≤ *M*, 1 ≤ *j* ≤ *N*. Since the sequencing depths (or library sizes) differ between experiments, we adjust read counts by applying the 'median ratio method' [3,8] to all experiments. More specifically, the adjusted read count ${x}_{ij}^{\hbox{'}}$ is calculated as the rounded value of *x*_*ij*_/*s*_*j*_, where *s*_*j*_ is the size factor in experiment *j* and computed as the median of all size factors calculated from individual sgRNA read counts:

1 $$ {s}_j= media{n}_i\left\{\frac{x_{ij}}{{\widehat{x}}_i}\right\} $$

where ${\widehat{x}}_i$ is the geometric mean of the read counts of sgRNA $i:{\widehat{x}}_i={\left({\prod}_{k=1}^N{x}_{ik}\right)}^{1/N}$.

#### Mean-variance modeling

To estimate the statistical significance of sgRNA abundance changes between conditions, we need to estimate the variance of the read counts within one condition (typically the control samples). Ideally, the variance can be estimated if there are enough replicates in one condition (for example, the approach used in SSMD [4,32]). However, the number of replicates is usually limited. We adopted the approaches used in edgeR [3,6,7] and DESeq [8,17] to model the variance. More specifically, we assume that the variance is a smooth function of the mean, and this function can be inferred using the mean and variance values of all sgRNAs. The simplest model is the Poisson model, which implies that the variance is equal to the mean. In many next-generation sequencing applications, however, the observed sample variance is substantially higher than the sample mean (over-dispersion) and the Poisson model substantially underestimates the true variance (Figure S1 in Additional file 1). To account for this over-dispersion, we assume the sample variance $\left({\widehat{\sigma}}^2\right)$ and sample mean $\left(\widehat{\mu}\right)$ satisfy the following empirical equation:

2 $$ {\widehat{\sigma}}^2=\widehat{\mu}+k{\widehat{\mu}}^b $$

or

3 $$ \log \left({\widehat{\sigma}}^2-\widehat{\mu}\right)= \log (k)+b \log \left(\widehat{\mu}\right),k\ge 0,b\ge 0 $$

This approach plugs in a consensus value for the individual sgRNA variances, thus effectively borrowing information between sgRNAs with similar read counts. To estimate the values of *k* and *b*, we calculate the sample mean $\left(\widehat{\mu}\right)$ and variance $\left({\widehat{\sigma}}^2\right)$ for each sgRNA normalized read count, and perform linear regression on $y= \log \left({\widehat{\sigma}}^2-\widehat{\mu}\right)$ against $x= \log \left(\widehat{\mu}\right)$. Finally, the parameters of the NB distribution can be determined from $\widehat{\mu}$ and ${\widehat{\sigma}}^2$ using the method of moments approach. More specifically, the parameters of the NB distribution *NB*(*r*, *p*) are calculated as:

$$ \begin{array}{l}p=1-\frac{\widehat{\mu}}{{\widehat{\sigma}}^2}\\ {}r=\frac{{\widehat{\mu}}^2}{{\widehat{\sigma}}^2-\widehat{\mu}}\end{array} $$

The above approach can be summarized as follows: sgRNA read counts are generated from a NB distribution, and the parameters of the NB distribution (that is, the mean and variance) for individual sgRNAs are determined by an empirical distribution in Equation 2. Note that similar models have been used in RNA-Seq differential expression tools (for example, edgeR [3,6,7] and DESeq [8,18]) to capture the mean and variance relationship of RNA-Seq read counts.

We also compared our mean-variance model with the model used in edgeR [4,7] and DESeq [8] (Figure S1 and Supplementary materials in Additional file 1). In edgeR (and later versions of DESeq), the variance is primarily determined by the squared mean (*b* = 2 in Equation 2) and only one parameter (*k*) needs to be estimated from the data. In the original DESeq paper, the variance is determined by the smoothed function *f* of the mean, where *f* is learned empirically from the data. (Notice that *f* does not have to be a quadratic function, as the NB assumption is not used in this step). The edgeR model using a common disperion value has a better fit for the variances for samples with larger *μ* but underestimates the variance for samples with smaller *μ* (Figure S1 in Additional file 1). This increases the number of significant selected sgRNAs for smaller *μ* where the variance estimates are less reliable. (Note that different normalization methods may also affect the performance of different algorithms; see Section B of Supplementary materials in Additional file 1 for more details).

#### sgRNA test and ranking

In this step, we test whether the read count difference of each sgRNA in two conditions (for example, in CRISPR/Cas9-treated samples versus control samples) is significant. We assume that the read count *x*_*iA*_ of sgRNA *i* in condition A follows a NB distribution:

$$ {x}_{iA}\sim NB\left({\mu}_{iA},{\sigma}_{iA}^2\right) $$

where *μ*_*iA*_ and ${\sigma}_{iA}^2$ are the mean and variance of the NB distribution, respectively. ${\sigma}_{iA}^2$ is adjusted using the mean-variance model learned from the previous step.

For a set of read counts of sgRNA *i* with replicates in two conditions A and B, we would like to test whether the read count is significantly different between the conditions. We first calculate the mean *μ*_*iA*_ and adjusted variance ${\sigma}_{iA}^2$ of condition A (typically the control condition) using the mean-variance model. After that, for the mean of read counts *μ*_*iB*_ of sgRNA *i* in condition B, we calculated the tail probability that the null NB distribution generates a read count that is more extreme than *μ*_*iB*_:

4 $$ p=\left\{\begin{array}{ll}{\displaystyle {\sum}_{x>{\mu}_{iB}}NB\left(x\left|{\mu}_{iA},{\sigma}_{iA}^2\right.\right),}\hfill & {\mu}_{iB}>{\mu}_{iA}\hfill \\ {}{\displaystyle {\sum}_{x<{\mu}_{iB}}NB\left(x\left|{\mu}_{iA},{\sigma}_{iA}^2\right.\right),}\hfill & {\mu}_{iB}<{\mu}_{iA}\hfill \end{array}\right. $$

Where $NB\left(x\Big|{\mu}_{iA},{\sigma}_{iA}^2\right)$ is the probability mass function (PMF) of a read count *x* from the NB distribution with mean *μ*_*iA*_ and variance ${\sigma}_{iA}^2$. This is the statistical significance of sgRNA *i* in two conditions. We provide two one-sided *P*-values to test whether sgRNA is positively selected (*μ*_*iB*_ > *μ*_*iA*_) or negatively selected (*μ*_*iB*_ < *μ*_*iA*_).

If there are no replicates in condition A, we estimate the mean and variance from all samples (in conditions A and B). This approach assumes that the majority of the sgRNAs have no effect on selection, which may not be true in some scenarios. Consequently, if there are no replicates, MAGeCK may be less sensitive as it overestimates the variance in one condition.

#### Gene test and ranking using modified robust rank aggregation (α-RRA)

A gene is considered essential if many of the sgRNAs targeting this gene rank near the top of the sgRNA list. To identify genes with a significant fraction of sgRNAs ranked near the top of the sgRNA list, which is sorted by NB *P*-values, we employed the RRA algorithm proposed by Kolde *et al.* [16]. Suppose *M* sgRNAs are included in the experiment, and *R* = (*r*_1_, *r*_2_, …, *r*_*n*_) is the vector of ranks of *n* sgRNAs targeting a gene (*n* < < *M*, *r*_*i*_ ≤ *M* where *i* = 1, 2, …, *n*). We first normalize the ranks into percentiles *U* = (*u*_1_, *u*_2_, …, *u*_*n*_), where *u*_*i*_ = *r*_*i*_/*M* (*i* = 1, 2, …, *n*). Under null hypotheses where the percentiles follow a uniform distribution between 0 and 1, the *k*th smallest value among *u*_1_, *u*_2_, …, *u*_*n*_ is an order-statistic which follows a beta distribution *B*(*k*, *n*, + 1 − *k*). RRA computes a *P*-value ρ_*k*_ for the *k*th smallest value based on the beta distribution. The significance score of the gene, the ρ value, is defined as ρ = min(*p*_1_, *p*_2_, …, *p*_*n*_).

We note that, when the sgRNAs targeting a gene concentrate in the middle of the sgRNA ranked list (that is, they have no effect on selection), RRA also computes a significant *P*-value for that gene and introduces false positives. This is because the assumption of uniformity is not necessarily satisfied in real applications. This is also a limitation of the frequently used Kolmogorov-Smirnov (KS) test. To solve this problem, we modified RRA by redefining the ρ value as follows. We first select the top ranked α% sgRNAs if their negative binomial *P*-values are smaller than a threshold (for example, 0.05). If *j* of the *n* sgRNAs targeting a gene are selected, then the modified ρ value is defined as ρ = min(*p*_1,_*p*_2_, …, *p*_*j*_), where *j* ≤ *n*. The modified RRA method, named α-RRA, can efficiently remove the effect of insignificant sgRNAs in the assessment of gene significance.

To compute a *P*-value based on the ρ values, we performed a permutation test where the sgRNAs are randomly assigned to genes (the numbers of sgRNAs targeting each gene remain unchanged). By default, 100 × *n*_*g*_ permutations are performed, where *n*_*g*_ is the number of genes. We then compute the FDR from the empirical permutation *P*-values using the Benjamini-Hochberg procedure [33].

#### Pathway test and ranking using α-RRA

We tested the enrichment of pathways based on the rankings of the genes using α-RRA, using the same approach we used to test genes. The pathway annotations include the KEGG canonical pathways [34] (181 pathways) database and the REACTOME pathway database [35] (676 pathways). We downloaded the annotations from GSEA MSigDB version 4.0 [36].

### Computational evaluation

#### Running RIGER

RIGER was originally designed to identify essential genes in genome-scale shRNA screens using microarray technology [14]. To accommodate the input requirements of RIGER, we median-normalized (the same as the first step of MAGeCK) and log2 transformed read counts from CRISPR/Cas9 knockout screens. We ran the latest version of RIGER (0.1 beta) as specified in the paper [14] and website [37]. Default RIGER parameters were used in all experiments, except that we set the number of permutations to 100,000 to get a more precise *P*-value. The results were ranked by the *P*-values of the genes.

#### Running RSA

RSA is an algorithm to rank essential genes based on the activity of siRNA knock-downs [15]. RSA ranks siRNAs by their fold enrichment. To accommodate the input requirements of RSA, we median-normalized the read counts from the CRISPR/Cas9 knockout screens (the same as the first step of MAGeCK). We defined the fold enrichment for each sgRNA as (Mean read counts in treatment samples)/(Mean read counts in control samples). We downloaded the latest version of RSA (v1.3) from the website [38]. For the negative selection experiments, we used the default parameters. For the positive selection experiments, we used the following parameters: -r (reverse picking), -u 1.0e8 (the upper bound of fold enrichment), -l 1 (the lower bound of fold enrichment).

#### Running edgeR and DESeq

We downloaded the latest versions of edgeR (v3.6.2) and DESeq (v1.16.0) from R Bioconductor [39]. When there were multiple replicates for one condition, we ran both DESeq and edgeR with default parameters. For edgeR, we estimated the common dispersion (using the estimateCommonDisp() function), and then estimated the tag-wise dispersion (using the estimateTagwiseDisp() function), as is indicated by the manual. If there were no replicates in one condition, we passed the following parameters to the dispersion estimation function in DESeq: method = 'blind' (ignore sample labels by treating all samples as replicates), sharingMode = 'fit-only'(use only the fitted values as the dispersion values), fitType = 'local' (use the local fit function as is described in the DESeq paper). For edgeR, we only estimated the common dispersion (using the estimateCommonDisp function).

#### sgRNA down-sampling

In the leukemia dataset, each gene was targeted by 10 predesigned sgRNAs (ribosomal genes are targeted by >30 sgRNAs). This dataset allowed us to compare MAGeCK with RIGER and RSA by using fewer targeting sgRNAs per gene. Using this dataset, we down-sampled the number of sgRNAs per gene to 10, 8, 6, 4, 2 and compared the results of the three algorithms. For evaluation, we used each algorithm to identify the same number of top-ranked (5%) genes separately using all sgRNAs. The intersection of these three lists of top-ranked genes yielded 188 genes, which we used as 'reference' genes to evaluate the performance of the different methods using fewer sgRNAs per gene.

#### Sequencing read down-sampling

We down-sampled the sequencing depth to evaluate the performance of MAGeCK. Initially we down-sampled reads to the minimum sequencing depth of all of the samples in each dataset (32 million in the leukemia dataset and 17.5 million in the melanoma dataset). Subsequently, we sampled different numbers of reads and evaluated the performance of MAGeCK.

#### Running on RNAi screening data

The pooled shRNA screen was performed in a previous study to identify genes whose knockdown confers resistance to PLX in A375 cells [17]. The screening results of RIGER were provided in the original paper, and we ran both MAGeCK and RSA from the shRNA rankings provided by RIGER. For MAGeCK, we provided the rankings of the shRNA to the RRA algorithm in MAGeCK with the threshold (α) set to be 0.05.

### Availability

The source code of MAGeCK is freely available at [40] under the 3-clause Berkeley Software Distribution (BSD) open-source license.

The datasets used in this paper, including the leukemia, melanoma and ESC datasets, are presented in Additional file 4.

## Additional files

Additional file 1: **Supplementary materials and Figures S1 to S7.**

Additional file 2: **Tables S1 to S10.**

Additional file 3: **Tables S11 to S18.**

Additional file 4: **Raw read counts of the leukemia, melanoma and ESC datasets.**

## Abbreviations

CRISPR: clustered regularly interspaced short palindromic repeats
DMSO: dimethyl sulfoxide
ESC: embryonic stem cell
FDR: false discovery rate
GeCKO: Genome-scale CRISPR/Cas9 knockout
KEGG: Kyoto Encyclopedia of Genes and Genomes
MAGeCK: Model-based Analysis of Genome-wide CRISPR/Cas9 Knockout
NB: negative binomial
PLX: vemurafenib
RIGER: RNAi Gene Enrichment Ranking
RNAi: RNA interference
RRA: robust ranking aggregation
RSA: Redundant siRNA Activity
sgRNA: single-guided RNA
shRNA: short hairpin RNA
siRNA: short interfering RNA
