# Supplementary material for: MAGeCK enables robust identification of essential genes from genome-scale CRISPR/Cas9 knockout screens
Source: Genome Biol. 2014 Dec 5;15(12):554. doi: 10.1186/s13059-014-0554-4 (PMC4290824; doi:10.1186/s13059-014-0554-4)
Supplement: Supplementary file 1 — Supplementary materials and Figures S1 to S7. [file 13059_2014_554_MOESM1_ESM.pdf]

Supplementary Materials

A. Comparing MAGeCK with edgeR, DESeq and baySeq

We compared the performance of MAGeCK with edgeR, DESeq and baySeq, three popular Negative Binomial-based algorithms for differential analysis of RNA-Seq. There are two different types of comparisons: comparisons between control samples or experimental replicates, and comparisons between treatment samples and proper control samples. For the first comparison, ideally no significant sgRNAs should be detected as the comparison is performed between control samples or replicates of the same condition. For the second comparison, a reasonable number of sgRNAs should be detected. (It is worth noting that the second comparison is not meant to demonstrate the superiority of one method over another, as the numbers of true positives are unknown.)

Table M1 depicts the numbers of significantly selected sgRNAs, using a False Discovery Rate (FDR) of 1% or 5%, between the different comparisons (the numbers of sgRNAs with unadjusted p-values less than 0.001 and 0.01 are shown in Table M2). MAGeCK has a lower FDR rate when comparing between controls or replicates than edgeR and DESeq, while the numbers of significantly selected sgRNAs between treatment/controls are similar. The p-values reported by baySeq are conservative for all comparisons.

Comparison	Leukemia dataset (HL-60 control vs. KBM7 control)		Melanoma dataset (14d control rep.1 vs. rep.2)		Melanoma dataset (14d PLX rep. 1 vs. rep. 2)		ESC dataset (ESC rep.1 vs. rep.2)	
FDR	1%	5%	1%	5%	1%	5%	1%	5%
MAGeCK	22	27	1	1	62	68	1	2
edgeR	699	926	71	201	95	170	0	0
DESeq	33	81	6	9	117	176	8	26
baySeq	0	0	0	0	0	0	0	0
Poisson	27867	30128	44	82	1042	1190	39564	42094
Comparison	Leukemia dataset (HL-60, KBM7 treatment vs. control)		Melanoma Dataset (7d PLX treatment vs. control)		Melanoma Dataset (14d PLX treatment vs. control)		ESC dataset (ESC vs. plasmid)	
FDR	1%	5%	1%	5%	1%	5%	1%	5%
MAGeCK	1840	2347	551	652	8012	8601	71	109
edgeR	1369	2224	993	1961	11631	18237	128	214
DESeq	1425	2163	428	717	6705	10869	83	156
baySeq	10	10	10	10	10	10	4	10
Poisson	47181	48688	5042	5999	27227	29141	10163	11918

Table M1. The number of significant sgRNAs, using a False Discovery Rate (FDR) of 1% or 5%, between control samples or between experiment replicates (top 6 rows), and between treatment vs. control samples (bottom 6 rows). In the control vs. control or replicate vs. replicate comparisons, if the null model fits the data, the numbers should be (close to) zero.

Comparison	Leukemia dataset (HL-60 control vs. KBM7 control) Total: 73151		Melanoma dataset (14d control rep.1 vs. rep.2) Total: 64076		Melanoma dataset (14d PLX rep. 1 vs. rep. 2) Total: 64076		ESC dataset (ESC rep.1 vs. rep.2) Total: 87437	
p value	0.001	0.01	0.001	0.01	0.001	0.01	0.001	0.01
MAGeCK	337	1235	60	302	73	152	66	672
edgeR	1003	1788	470	1492	384	1267	139	2358
DESeq	277	1129	126	706	298	857	200	1071
baySeq	NA	NA	NA	NA	NA	NA	NA	NA
Poisson	42369	48680	1669	5166	4182	8456	55406	61747
Comparison	Leukemia dataset (HL-60, KBM7 treatment vs. control) Total: 73151		Melanoma Dataset (7d PLX treatment vs. control) Total: 64076		Melanoma Dataset (14d PLX treatment vs. control) Total: 64076		ESC dataset (ESC vs. plasmid) Total: 87437	
p value	0.001	0.01	0.001	0.01	0.001	0.01	0.001	0.01
MAGeCK	8943	15180	2011	4237	13496	19179	703	1245
edgeR	1852	3796	1764	4378	10481	16976	427	1490
DESeq	1987	3592	845	2218	6642	11386	325	754
baySeq	NA	NA	NA	NA	NA	NA	NA	NA
Poisson	55872	59565	15659	22887	39527	44942	27408	37214

Table M2. The numbers of detected significant sgRNAs, using p value cutoff of 0.001 or 0.01, between control samples or experiment replicates (top 6 rows), and between treatments vs. control samples (bottom 6 rows). The numbers for baySeq are unavailable, as baySeq only provides adjusted p values. In the control vs. control or replicate vs. replicate comparisons, if the null model fits the data, then the proportion of sgRNAs that are significant should be equal to the p-value cutoff. For example, a good null model should detect approximately 732 significant sgRNAs with p-value smaller than 0.01 in leukemia dataset (732=73151*0.01).

We compared the effect of 3 different variance-fitting models on gene rankings in Table M3. We applied the alpha-RRA algorithm on the sgRNA list ranked by 3 models. The same comparisons used in Table M1 are used here. The results demonstrate that three different

models produce similar results in different comparisons. This is not surprising because MAGeCK adopts a similar approach to edgeR and DESeq. edgeR has a slightly higher FDR than MAGeCK and DESeq, consistent with its higher FDR at the sgRNA level as shown in Table M1.

Comparison	Leukemia dataset (HL-60 control vs. KBM7 control)		Melanoma dataset (14d control rep.1 vs. rep.2)		Melanoma dataset (14d PLX rep. 1 vs. rep. 2)		ESC dataset (ESC rep.1 vs. rep.2)	
FDR	5%	25%	5%	25%	5%	25%	5%	25%
MAGeCK	0	0	0	0	0	0	0	0
edgeR	0	5	0	1	0	0	0	9
DESeq	0	0	0	0	0	0	1	1
Comparison	Leukemia dataset (HL-60, KBM7 treatment vs. control)		Melanoma Dataset (7d PLX treatment vs. control)		Melanoma Dataset (14d PLX treatment vs. control)		ESC dataset (ESC vs. plasmid)	
FDR	5%	25%	5%	25%	5%	25%	5%	25%
MAGeCK	335	602	40	130	12	34	310	596
edgeR	310	522	43	139	10	27	342	666
DESeq	315	557	58	91	11	35	367	744

Table M3.The number of significant genes using a False Discovery Rate (FDR) of 1% or 5%, between control samples or experiment replicates (top 5 rows), and between treatment and control samples (bottom 5 rows). sgRNA rankings are produced by different algorithms ranked by p value, and gene rankings and their p values are assigned by alpha-RRA.

B. Comparing different normalization methods

We compared different normalization methods in two CRISPR/Cas9 knockout screening datasets in Figure M**1**. Two normalization methods are compared, including median normalization (“median”) and total read count normalization (“total”). Using different normalizations, the distribution of the read counts of significant sgRNAs (FDR=1%) is plottedand compared with the mean read count distribution of all sgRNAs (“all”, black). An unbiased method should have a distribution of sgRNA read counts that is similar to the distribution of all sgRNAs. Figure M**1** shows that the differences are subtle for the leukemia dataset; but in the melanoma dataset where a few sgRNAs have very large read counts, “total” normalization will prefer sgRNAs with higher read-counts. In contrast, the distribution after “median” normalization is closer to the distribution of all sgRNAs.

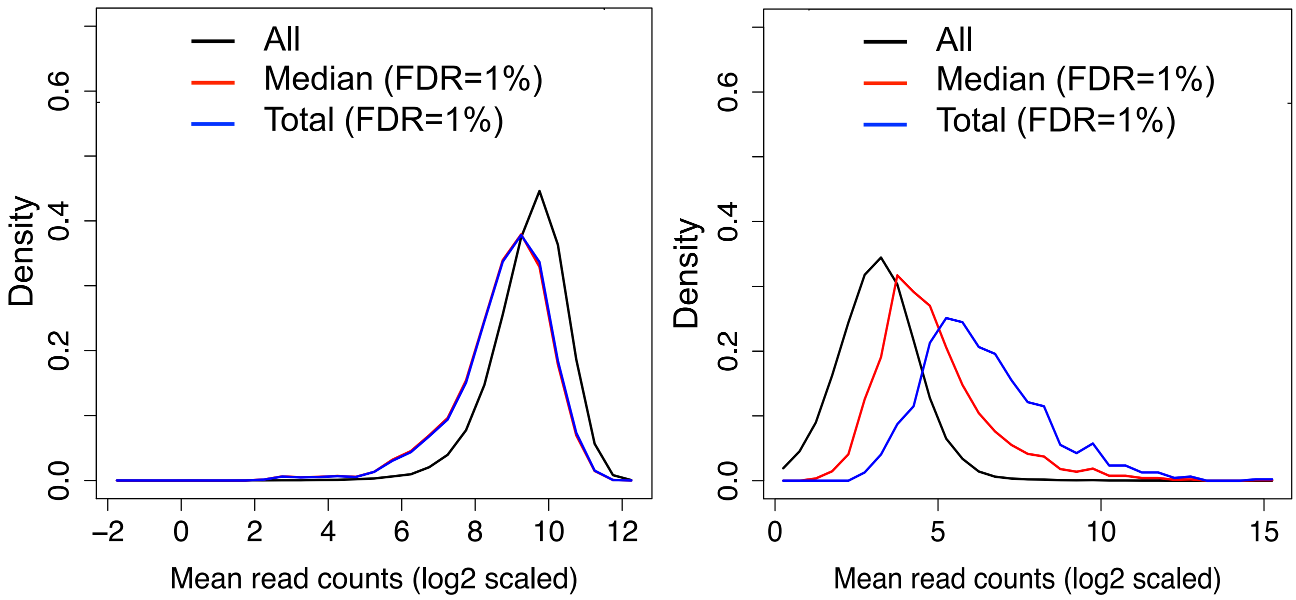

Figure M1. A comparison of different normalization methods in the leukemia dataset (left) and melanoma dataset (right).

In DESeq and edgeR, the normalization step is performed by size factor estimation. Differences in size factor estimation may contribute to the differences in performance shown in Table M1. Table M4 compares the effect of different size factors on the control and replicate comparisons. Here, the default size factor estimation of edgeR, DESeq and MAGeCK are compared. To use MAGeCK size factor estimation in edgeR and DESeq, we first normalize read counts by MAGeCK, and then run edgeR/DESeq without any size factor estimation. Table M4 shows that the normalization method difference may partially explain the improved performance of MAGeCK, but the improvement differs across datasets. For example, substantial improvements in the false discovery rate can be achieved using MAGeCK's normalization method in the melanoma dataset, but such improvements are not seen in the other datasets (i.e. the leukemia and ESC datasets).

Comparison	Leukemia dataset (HL-60 control vs. KBM7 control)		Melanoma dataset (14d control rep.1 vs. rep.2)		Melanoma dataset (14d PLX rep. 1 vs. rep. 2)		ESC dataset (ESC rep.1 vs. rep.2)	
FDR	1%	5%	1%	5%	1%	5%	1%	5%
edgeR (with default size factor estimation)	699	926	71	201	95	170	0	0
edgeR (with MAGeCK size factor estimation)	771	926	2	3	76	90	0	0
DESeq (with default size factor estimation)	33	81	6	9	117	176	8	26
DESeq (with MAGeCK size factor estimation)	40	91	2	3	47	52	8	28

Table M4. The effect of different size factor estimation methods on MAGeCK, DESeq and edgeR.

C. Choosing the value of α in α-RRA

In the α-RRA algorithm of MAGeCK, the value of α can be specified by the user and the default value is 0.05. If $\alpha = 1$, then the α-RRA algorithm is the same as the original RRA algorithm. To study the effect of different choices of α, we used the leukemia dataset and recorded the numbers of significantly selected genes using different values of α (the results from other datasets are similar). We used two different comparisons: HL-60 control vs. KBM7 control (to evaluate the false discovery rate) and HL-60, KBM7 treatment vs. control (to evaluate the sensitivity. Figure M2 records the numbers of essential genes that are statistically significant in different comparisons at different FDR cutoffs (0.01, 0.05 and 0.25). The false discovery rate of the α-RRA algorithm is robust against different choices of α, as there are no significantly selected genes detected by comparing between controls. For comparisons between treatment and control samples (where some essential genes like ribosomal genes are expected), the number of genes increases slightly as α increases from FDR=0.01 to 0.05, and substantially increases as α increases to FDR=0.25.

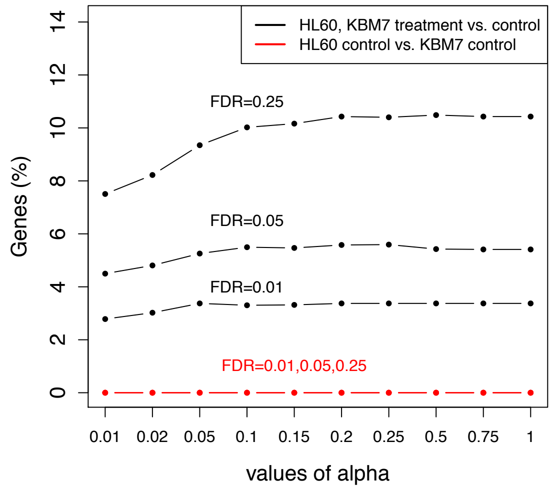

Figure M2. The number of essential genes that are statistically significant in the leukemia dataset, using different values of α and different FDR cutoffs.

The MAGeCK algorithm is generally robust against the choice of α in terms of genes with lower FDR cutoffs, but for higher FDR cutoffs, the number of significantly selected genes increases if a larger value of α is set. We use $\alpha = 0.05$ as the default value because it has a reasonable level of sensitivity in treatment vs. control comparisons in all datasets, and it assigns higher p values to some "negative control" genes with many targeting sgRNAs (see Table M5).

Genes	# targeting sgRNAs	$\alpha = 0.05$		$\alpha = 1.00$	
		p value	FDR	p value	FDR
RPS4Y2	49	6.68e-4	0.30	1.94e-5	0.08
RPS4Y1	38	1.16e-3	0.35	1.16e-3	0.29

Table M5. The p value assignments of two non-essential genes, *RPS4Y1/2* (located in Y chromosome), using different values of α.

Supplementary Figures

Supplementary Figure 1. Comparisons of different variance fitting models in the leukemia dataset (top left), melanoma dataset (top right), and ESC dataset (bottom). The raw variance together with the fitted curves of the Poission, MAGeCK, edgeR and DESeq models are shown. In the leukemia dataset, two control samples from HL-60 and KBM7 cell lines are used; in the melanoma dataset, two control samples from 14 days are used; and in the ESC dataset, two CRISPR/Cas9 knockout treated replicates are used.

Supplementary Figure 2. The normalized read counts of 10 sgRNAs targeting the gene FAM163B in the KBM7 cell line of the leukemia dataset. The first sgRNA shows a 42-fold decrease of abundance in the CRISPR/Cas9 knockout treated sample compared with the control sample, and ranks highly in the sgRNA list (e.g. ranked 67 by MAGeCK, FDR=4.67E-45). However, the abundances of the other 9 sgRNAs for this gene do not change as much (and often change in the opposite direction). This suggests that the gene FAM163B may not be a negatively selected gene, and the abundance change of the first sgRNA may be explained by other factors (such as off-target effects). MAGeCK reports the ranking of the gene FAM163B as 768, with FDR=0.35.

Supplementary Figure 3. Cumulative read count distributions of sgRNAs in the leukemia (left) and melanoma datasets (right). The x axis is the index of the sgRNAs, sorted by their read counts, and the y axis is the cumulative fraction of the read counts. In the leukemia dataset, the raw read counts of KBM7 before (KBM7 initial) and after sgRNA transfection (KBM7 final) are shown; in the melanoma dataset, read counts from the 14 days PLX treatment (14d PLX) and the corresponding 14 days control samples are shown.

Supplementary Figure 4. The distribution of p values calculated using a Poisson model (A, B) and using MAGeCK (C, D) from the leukemia dataset, where two controls are compared with each other. 20 equally distributed bins of p-values are used, and the distributions before (A, C) and after multiple comparison adjustment (B, D) are shown. Ideally no significant genes should be detected as the comparison is performed between control samples or replicates of the same condition. The results from edgeR and DESeq are similar to MAGeCK.

Supplementary Figure 5. The mean read count distribution of the top 5% of sgRNAs from different ranking algorithms in HL-60 and KBM7 treatment vs. control comparisons in the leukemia dataset. As in Supplementary Figure 2, an unbiased method should give a distribution of sgRNA read counts that resembles the distribution of all sgRNAs. The top-ranked sgRNAs using the fold change approach (used by RSA) are biased towards those with lower read counts.

Supplementary Figure 6. The gene p-value distribution from three different methods using the leukemia dataset (HL-60 control vs. KBM7 control). RIGER p-values are biased toward high values (>0.8), while the numbers of low p-values from RSA is higher than MAGeCK.

Supplementary Figure 7. The read distribution of sgRNAs with FDR<1% in 14-day PLX treatment (right) and the corresponding control sample (left). 1M down-sampled reads are used. On the left, the significant sgRNAs have only 10% of the reads while on the right the fraction is 64%.

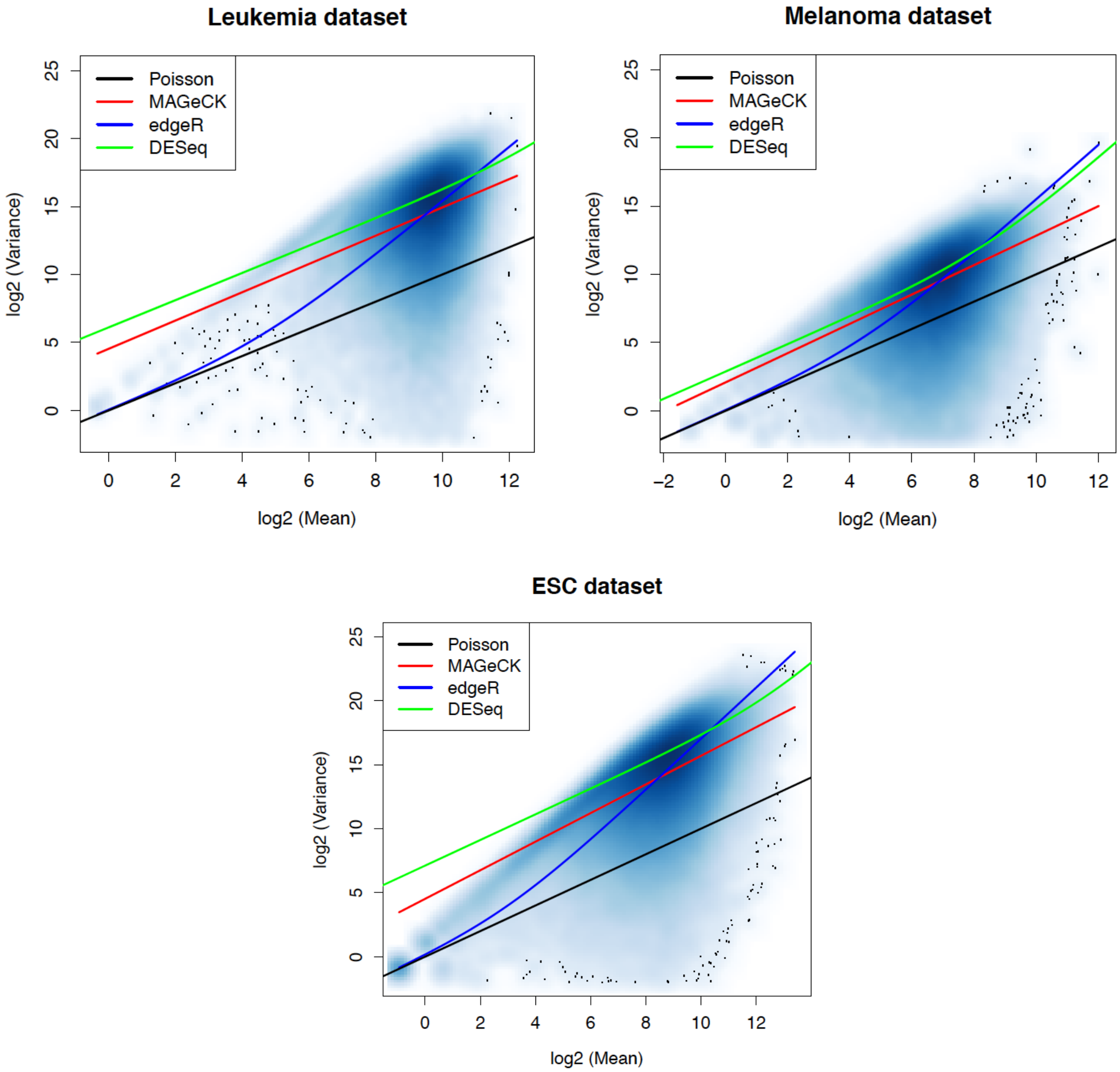

Supplementary Figure 1. Comparisons of different variance fitting models in the leukemia dataset (top left), melanoma dataset (top right), and ESC dataset (bottom). The raw variance together with the fitted curves of the Poission, MAGeCK, edgeR and DESeq models are shown. In the leukemia dataset, two control samples from HL-60 and KBM7 cell lines are used; in the melanoma dataset, two control samples from 14 days are used; and in the ESC dataset, two CRISPR/Cas9 knockout treated replicates are used.

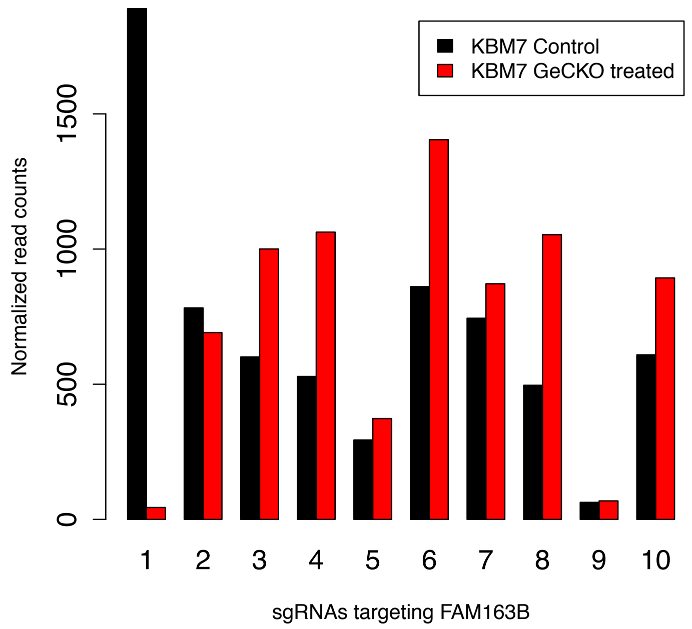

Supplementary Figure 2. The normalized read counts of 10 sgRNAs targeting the gene FAM163B in the KBM7 cell line of the leukemia dataset. The first sgRNA shows a 42-fold decrease of abundance in the CRISPR/Cas9 knockout treated sample compared with the control sample, and ranks highly in the sgRNA list (e.g. ranked 67 by MAGeCK, FDR=4.67E-45). However, the abundances of the other 9 sgRNAs for this gene do not change as much (and often change in the opposite direction). This suggests that the gene FAM163B may not be a negatively selected gene, and the abundance change of the first sgRNA may be explained by other factors (such as off-target effects). MAGeCK reports the ranking of the gene FAM163B as 768, with FDR=0.35.

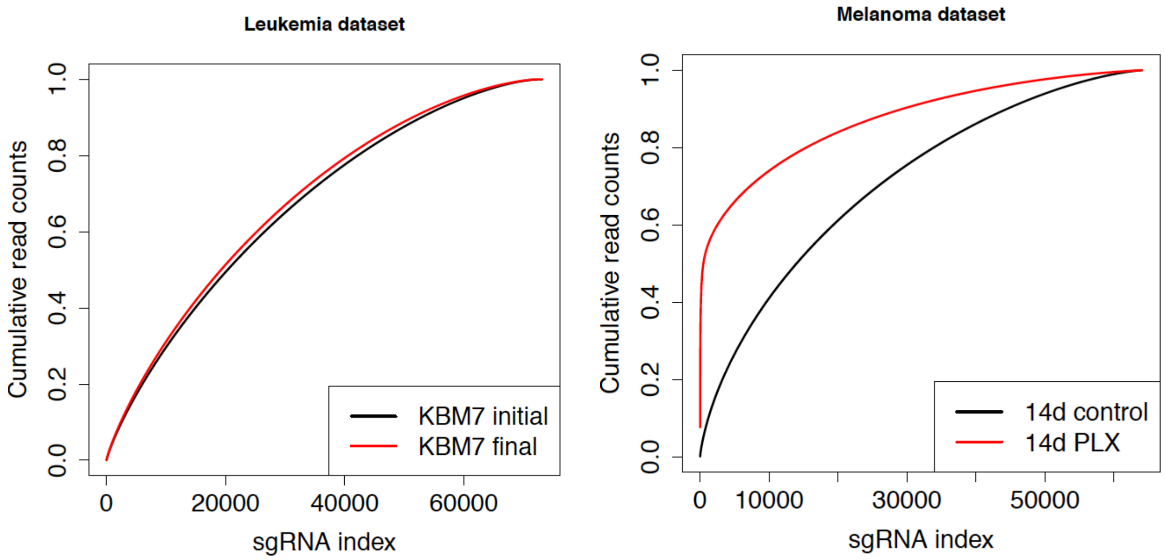

Supplementary Figure 3. Cumulative read count distributions of sgRNAs in the leukemia (left) and melanoma datasets (right). The x axis is the index of the sgRNAs, sorted by their read counts, and the y axis is the cumulative fraction of the read counts. In the leukemia dataset, the raw read counts of KBM7 before (KBM7 initial) and after sgRNA transfection (KBM7 final) are shown; in the melanoma dataset, read counts from the 14 days PLX treatment (14d PLX) and the corresponding 14 days control samples are shown.

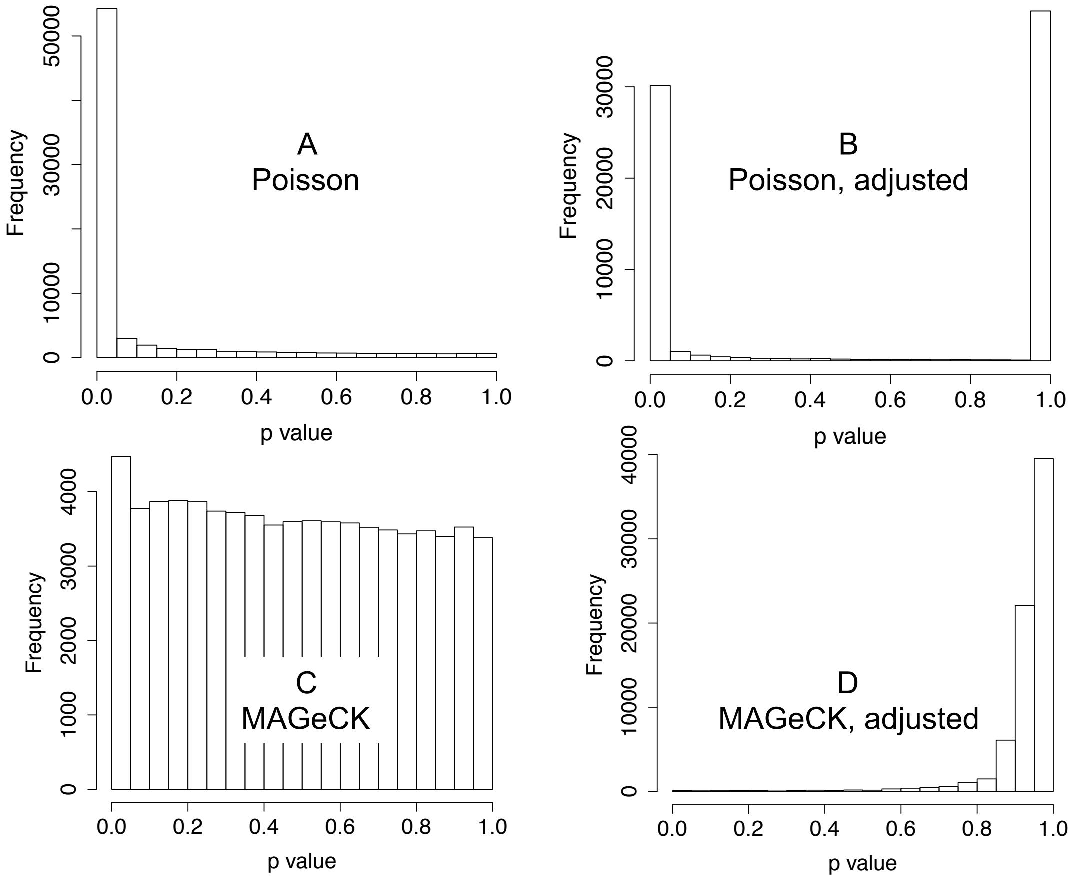

Supplementary Figure 4. The distribution of p values calculated using a Poisson model (A, B) and using MAGeCK (C, D) from the leukemia dataset, where two controls are compared with each other. 20 equally distributed bins of p-values are used, and the distributions before (A, C) and after multiple comparison adjustment (B, D) are shown. Ideally no significant genes should be detected as the comparison is performed between control samples or replicates of the same condition. The results from edgeR and DESeq are similar to MAGeCK.

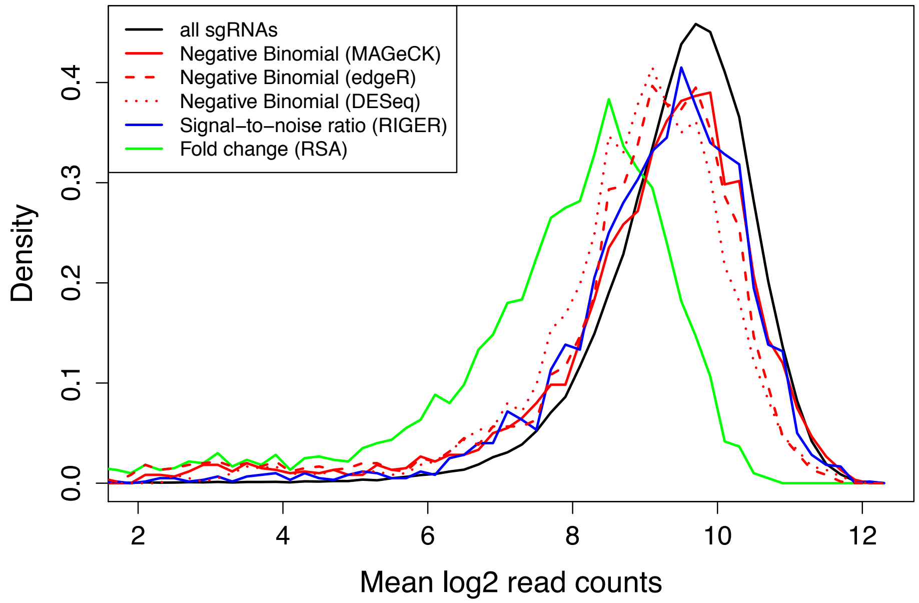

Supplementary Figure 5. The mean read count distribution of the top 5% of sgRNAs from different ranking algorithms in HL-60 and KBM7 treatment vs. control comparisons in the leukemia dataset. As in 0, an unbiased method should give a distribution of sgRNA read counts that resembles the distribution of all sgRNAs. The top-ranked sgRNAs using the fold change approach (used by RSA) are biased towards those with lower read counts.

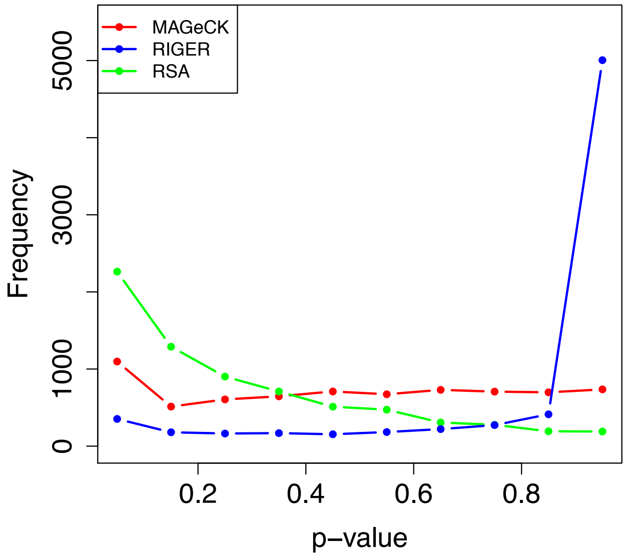

Supplementary Figure 6. The gene p-value distribution from three different methods using the leukemia dataset (HL-60 control vs. KBM7 control). RIGER p-values are biased toward high values (>0.8), while the numbers of low p-values from RSA is higher than MAGeCK.

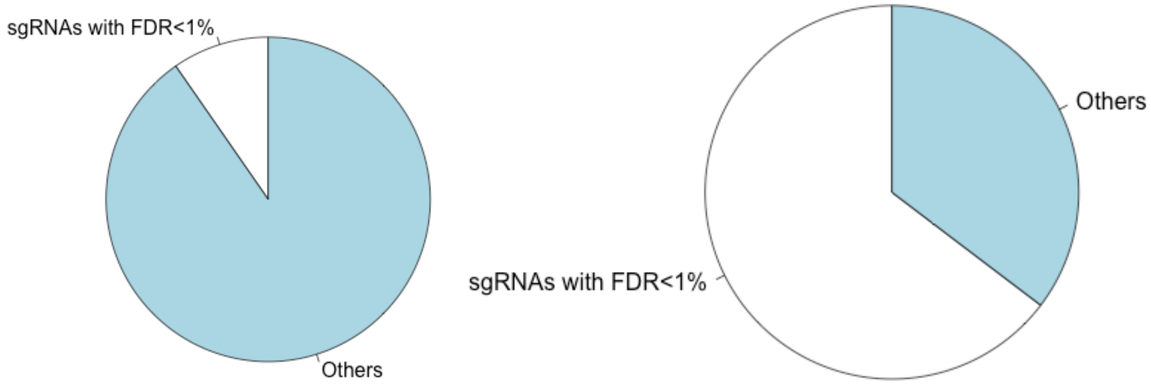

Supplementary Figure 7. The read distribution of sgRNAs with FDR<1% in 14-day PLX treatment (right) and the corresponding control sample (left). 1M down-sampled reads are used. On the left, the significant sgRNAs have only 10% of the reads while on the right the fraction is 64%.
